# Isolation and characterization of epithelial cells and fibroblasts from the human penile urethra

**DOI:** 10.3389/fbioe.2025.1713156

**Published:** 2025-12-10

**Authors:** David Brownell, Elissa Elia, Félix-Antoine Pellerin, Stéphane Chabaud, Sébastien Larochelle, Véronique J. Moulin, Alexis Laungani, Stéphane Bolduc

**Affiliations:** 1 Centre de Recherche en Organogénèse Expérimentale/LOEX, CHU de Québec-Université Laval Research Center (Regenerative Medicine Division), Université Laval, Quebec City, QC, Canada; 2 GrS Montréal, Montreal, QC, Canada; 3 Division Science et Enseignement, Département de chirurgie plastique, Université de Montréal, Montreal, QC, Canada; 4 Department of Surgery, Université Laval, Quebec City, QC, Canada

**Keywords:** primary cell isolation, penile urethra, dispase, thermolysin, urethral epithelial cells, urethral fibroblasts, urethral tissue engineering

## Abstract

**Introduction:**

Urethral strictures and hypospadias are common urological conditions for which autologous reconstruction remains challenging. Tissue engineering offers a promising alternative, yet current strategies often rely on heterotopic cell sources, potentially limiting functional integration. Here, we report the first isolation and characterization of epithelial and stromal cells from distinct regions of the human penile urethra: the spongy urethra and the proximal and distal fossa navicularis.

**Methods:**

Cells were isolated from the three regions of the penile urethra in 12 donors. Detailed characterization was performed for 3–4 donors, assessing yield, growth parameters, immunophenotype, and progenitor preservation. We evaluated 3-, 4-, and 6-mm biopsies to determine the minimal tissue size required for clinically relevant cell yields. Multiple enzymatic protocols were compared, using thermolysin or dispase II for epithelial-stromal separation, followed by collagenase ± elastase digestion for stromal cell recovery.

**Results:**

Cell extraction had a 100% success rate across all tested protocols. The combination of dispase and a 4-h collagenase/elastase digestion yielded the highest cell numbers and clonogenic potential. All biopsy sizes produced sufficient cells for tissue engineering.

**Discussion:**

These findings demonstrate the feasibility of harvesting high-quality, organ-specific autologous cells from minimal urethral biopsies. In addition to their clinical potential, these cells provide a foundation for preclinical disease modelling using patient-derived pathological cells, which are currently unavailable for in vitro studies of urethral disorders.

## 1 Introduction

Pathologies affecting the urogenital tract imply a significant impact on quality of life due to the psychosocial importance of the genitals (Zhu et al., 2012; Jin et al., 2022). Various pathologies can affect the human urethra, such as hypospadias, which affects 1 in 150 boys (Hypospadias, Birth Defects, CDC, n.d.). This is a congenital anomaly where the urethral meatus is located on the ventral side of the penis, implying complications related to urination and sexual intercourse (van der Horst and de Wall, 2017). As the world has modernized, the presence of endocrine disruptors in the environment has significantly increased, leading to an increase in hypospadias amongst other endocrine developmental disorders (Mattiske and Pask, 2021).

Recent studies have demonstrated differences in gene expression in boys with hypospadias (Qiao et al., 2012a; Qiao et al., 2012b; Inanc et al., 2023; Perske et al., 2023), however, there are currently no commercially available penile urethral cells, let alone from hypospadias patients. *In vitro* studies could help elucidate the underlying mechanisms involved in urethral disorders if cells were available.

Besides the important implications related to the *in vitro* evaluation of pathological cells from the penile urethra, tissue engineering is also a field with significant interest for treating individuals with hypospadias or other pathologies, such as urethral stricture or fistulas. When sourcing cells for tissue engineering, one must be mindful of physiological relevance. Currently, all cell-based tissue engineering constructs designed for urethral reconstruction rely on heterotopic cell sources (Culenova et al., n.d.). Common sources for engineered urethral constructs are from the bladder (Culenova et al., n.d.), as it is considered a urothelial tissue, or from the oral mucosa, as the environment in the mouth is often considered similar to that of the penile urethra. By grafting a tissue constructed using heterotopic cells, the importance of tissue specificity and the distinct role of the urethral epithelium are ignored. For example, the bladder is composed of a transitional epithelium, while the oral mucosa is made of a stratified squamous epithelium, which is not made to resist urine. Factors such as microbiome composition have been shown to be more important than once thought (Perez-Carrasco et al., 2021), further backing the importance of using organ-specific cells for tissue engineering.

The histology of the penile urethra was recently described in detail (Duval et al., 2025), filling this knowledge gap, hindering tissue engineers in reproducing the most physiologically relevant construct possible. It was demonstrated that there are 3 distinct zones within the penile urethra. In the Fossa Navicularis (FN), the distal portion presents a stratified squamous histology with glycogen rich apical cell layers, similar to the vagina. The glycogen presence promotes *lactobacillus* growth, creating an acidic environment which protects against infection (Nelson et al., 2012). The proximal FN is significantly thinner and lacks glycogen reserves. In the epithelium of the spongy urethra, a columnar pseudostratified aspect is observed.

When extracting epithelial cells for applications in tissue engineering, epithelial population quality, defined by the retention of progenitor cells, is vital for stem cell niche formation, and ultimate long term graft maintenance (Lavoie et al., 2013).

**FIGURE 1:**
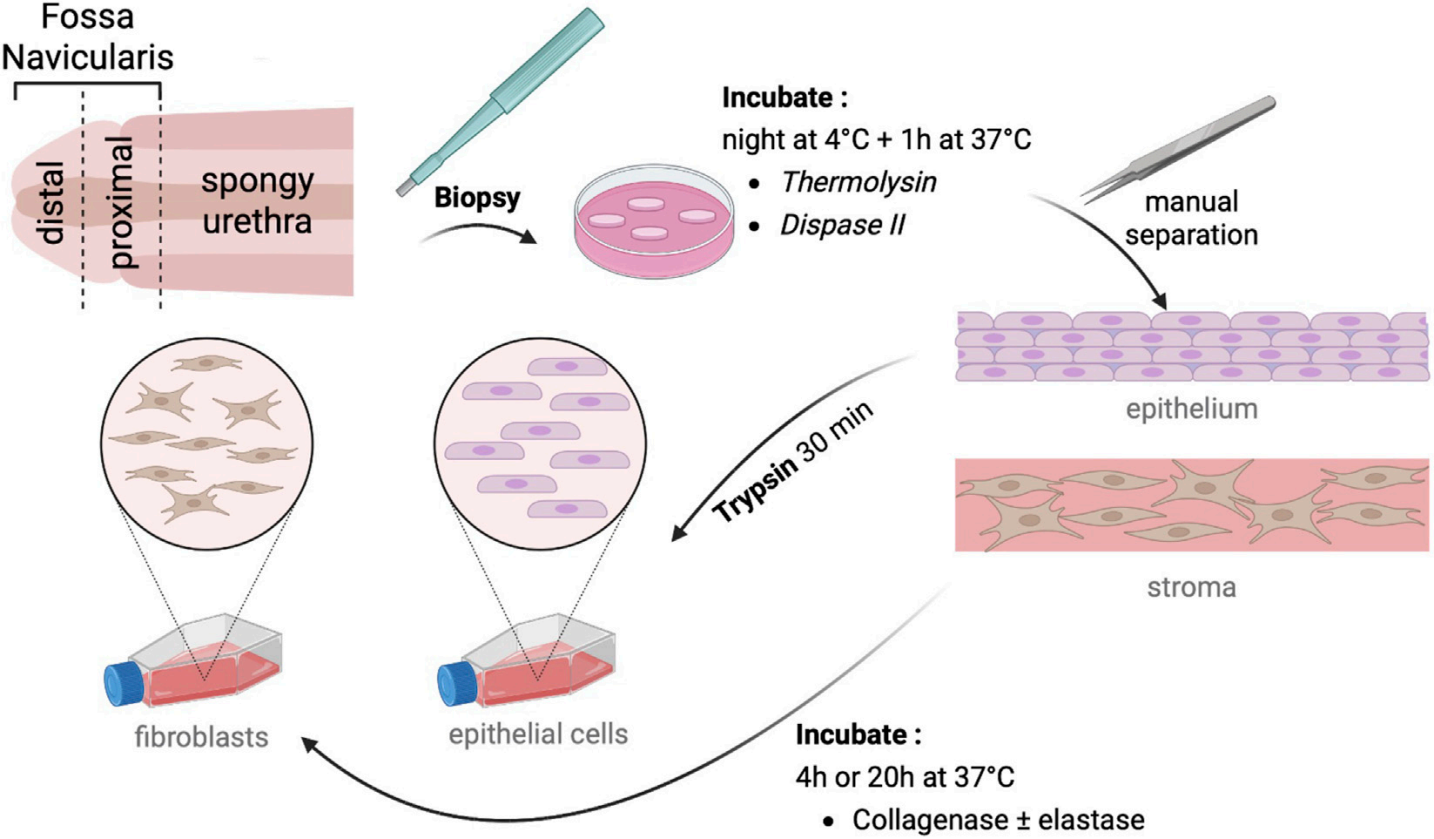
Description of cell isolation protocol. After biopsy collection, samples are incubated at 4 °C overnight, then at 37 °C for 1 h with thermolysin or dispase II. The epithelium was manually separated from the stroma and trypsin-treated for epithelial isolation. The stroma was digested with collagenase ± elastase to isolate fibroblasts. Cells were seeded in appropriate media for further characterization.

What is, to our knowledge, the first account of epithelial cell and fibroblast extraction from the human penile urethra is described here. Several extraction methods were compared (Figure 1), as yield is important when considering minimizing biopsy size in individuals requiring urethral reconstruction. Fibroblasts and epithelial cells were extracted from the three distinct regions of the penile urethra and each population was characterized by immunofluorescent labeling and compared to vaginal and bladder epithelial cells.

## 2 Materials and methods

### 2.1 Ethics statement

This study was conducted according to the Declaration of Helsinki and was approved by the institution’s committee for the protection of human participants (Comité d’éthique de la recherche du CHU de Québec-Université Laval, protocol number DR-002-1190). All patients provided informed written consent prior to biopsies.

### 2.2 Cell culture

Fibroblasts were grown in complete DMEM (cDMEM) (Dulbecco–Vogt modified Eagle’s medium (DMEM, Corning, Corning, NY, United States) with 10% fetal bovine serum (FBS, Avantor Seradigm FB Essence, Randor, PA, United States), 100 U/mL penicillin (Sigma-Aldrich, Saint Louis, MO, United States), and 25 μg/mL gentamicin (Schering, Pointe-Claire, QC, Canada). Epithelial cells were grown on dermal fibroblast feeder cells (foreskin fibroblasts growth arrested with 60 Gy γ-radiation) in complete DMEM-Ham’s F-12 media (cDH) (3:1 mix of DMEM and Ham’s F12 media (Gibco, Grand Island, NY, United States), supplemented with 5% FBS (GE Healthcare, Chicago, IL, United States), 24.3 μg/mL adenine (Corning), 5 μg/mL crystallized bovine insulin (Sigma-Aldrich), 1.1 μM hydrocortisone (Teva Canada Ltd., Scarborough, Canada), 0.212 μg/mL isoproterenol hydrochloride (Sandoz Canada, Boucherville, QC, Canada), 10 ng/mL epidermal growth factor (Austral Biologicals, San Ramon, CA, United States), 100 U/mL penicillin, and 25 μg/mL gentamicin). Cells were passaged at confluence using 0.05% trypsin/0.01% EDTA (Gibco) and cryopreserved in FBS containing 10% dimethyl sulfoxide (DMSO, Sigma-Aldrich) for later evaluation. Frozen cells were thawed in a 37° water bath and diluted 10X by slowly adding respective media. Cells were seeded at 1.67 × 10^5^/cm^2^.

### 2.3 Specimen retrieval

Twelve specimens were retrieved from transgender patients aged 19–77 years old undergoing gender affirming vaginoplasty. The specimens contained the entire penile urethra, surrounded by the corpus spongiosum. Most of the glans and a portion of the corpus cavernosum were still attached to the specimen. The tissues were dissected in a surgical theatre by slicing longitudinally along the middle of the ventral surface to expose the entire urethral mucosa. The mucosa was then dissected according to each extraction protocol.

### 2.4 Extraction using dispase II or thermolysin

A 5 mm strip was isolated from the urethral mucosa from either the distal or proximal FN, or the spongy urethra for 4 patients (22, 28, 41, 58 years old). The strips were cut in half and rinsed 3 times in phosphate-buffered saline (PBS) pH 7.4 containing 100 U/mL penicillin, 25 μg/mL gentamicin, and 0.5 μg/mL amphotericin B (Fisher Scientific, Ottawa, ON, Canada). Half of each strip was placed in a 2.5 U/mL Dispase II (Roche Diagnostics, Mannheim, Germany) solution or a 500 μg/mL Thermolysin (Sigma-Aldrich) solution diluted in extraction buffer (DMEM low glucose without phenol (Sigma-Aldrich)/20 mM HEPES/0.15% fatty acid-free bovine serum albumin (Proliant, Ankeny, United States)/pH 7.4)) and incubated overnight at 4 °C, followed by 1 h at 37 °C. Samples were cut into 3 mm wide strips with a scalpel in a biosecurity hood 4–7 h after collection due to transport from the clinic. At this time, samples were transferred from Falcon tubes to 60 mm Petri dishes to improve enzyme diffusion.

The epithelium was mechanically separated from the stroma using fine tweezers, then incubated for 30 min at 37 °C in a 0.05% trypsin/EDTA solution under gentle agitation. The trypsin was inhibited with an equal volume of cDH, the primary human urethral epithelial cells were then pelleted and seeded in three T75 flasks per biopsy (approximately 225 cm^2^ culture surface per 1 cm^2^ of biopsy). Cultures were passaged at confluence and cryopreserved for future analyses.

Primary human urethral fibroblasts were then extracted from the stromal compartment by incubation with 2.5 U/mL collagenase I/II GMP Grade (CustomBiotech Roche Diagnostics) in extraction buffer containing 1.5% BSA for 4 h at 37 °C under gentle agitation. The solution was then diluted with 2 volumes of DMEM. Cells were pelleted and seeded in 3 T75 flasks per biopsy. Cultures were passaged at confluence and cryopreserved for future analyses.

### 2.5 Clonogenicity evaluation

Epithelial cells were cultured up to passage 4, and clonogenicity was evaluated at each passage for the Dispase II and Thermolysin conditions. 1.67 × 10^5^ dermal feeder cells were seeded per T25 flask in cDH a week prior to colony forming efficiency evaluation. At epithelial cell passaging, half the media was removed from feeder cells, and 500 epithelial cells were seeded per flask in an equal volume of fresh cDH. Cells were cultured for 10 days, with one media change at day 5. The cells were fixed in a 3.7% neutral buffered formol solution and colonies were stained with a Nile blue A/rhodamine mixture (Sigma-Aldrich) for 15 min and then rinsed three times with tap water, and the flasks were air dried at room temperature. The flasks were scanned using a Typhoon Trio + scanner (GE Healthcare) with a 633 nm excitation laser and a 670BP30 filter and a resolution of 200 pixels/cm. ImageJ software (NIH, Bethesda, MD) was used to count colonies. Holoclones were considered ∅ ≥ 4.5 mm, meroclones as 1.5 mm < ∅ < 4.5 mm, and paraclones as ∅ ≤ 1.5 mm. Ratio paired t-tests were used to statistically evaluate differences in clonogenicity between extraction conditions.

### 2.6 Fibroblast extraction with elastase

To optimize fibroblast extraction, urethral mucosa biopsies from four patients of 19-, 35-, 39-, and 52-years-old were made using a 6 mm biopsy punch in technical triplicates, and biopsies were weighed for normalization. After removal of the epithelium using Dispase II, stromal compartments were digested with 2.5 U/mL collagenase I/II in extraction buffer containing DNase, either with or without 5 U/mL Elastase (Serva, Heidelberg, Germany). Digestion was carried out for either 4 h or 20 h (overnight). Cells were counted before seeding using a Coulter Z2 counter. One T75 and three wells of a 6-well plate were seeded per biopsy. When the first wells were 80% confluent, all wells were passaged and counted to evaluate yield. Statistical differences were evaluated using a 2-way ANOVA followed by a Tukey’s test.

### 2.7 Evaluation of yield per biopsy size

Biopsy punches of 3-, 4-, or 6-mm diameter were used to cut the urethral mucosa for cell isolation. Biopsies from 4 specific regions of 4 patients (19, 35, 39, and 52 years-old) were digested with Dispase II for epithelial cell isolation, then Collagenase I/II with DNase overnight for fibroblast isolation. Before the first seeding, cells were counted with a Coulter Z2 counter to evaluate cell yield. Statistical differences were evaluated using a 1-way ANOVA followed by a Tukey’s test.

### 2.8 Immunofluorescence staining

Epithelial cells and fibroblasts at P2 from the urethra, vagina, and bladder of patients aged 51, 32, and 3 years old, respectively, were passaged at confluence and seeded at 105 cells/cm^2^ (confluence) in 12-well plates containing 18 mm glass coverslips. Cells were cultured for 1 day, then rinsed 3X with PBS containing calcium and magnesium ions (PBS-IF). The cells were then treated with ice-cold (−20 °C) 100% methanol for 10 min at −20 °C and rinsed 3X with PBS-IF. Cells were then incubated at room temperature for 45 min with PBS-IF containing 1% BSA and respective dilutions of primary antibodies: MUC1 (1:200, sc-7313), CK10 (1:500, ab9025), AE1/AE3 (1:200, MABB412), CK18 (1:100, ARP03-61009), ΔNp63 (1:100, CST-67825), CK14 (1:1000, CLPRB-155p), vimentin (1:500, ab45939), ERβ (1:100, PA1-313). Cells were rinsed 3X, 2 min with PBS-IF, then incubated for 30 min at room temperature with PBS-IF, 1% BSA containing secondary antibodies: AF594 donkey anti-mouse (1:200, R37115), AF488 donkey anti-rabbit (1:200, A-21206) and Hoechst (5 mg/mL, Sigma). Cells were rinsed 3X, 2 min with PBS-IF and 2X, 2 min with distilled water. Coverslips were mounted on slides with mounting medium (PBS-glycerol-gelatin, pH 7.6). Samples were observed with a Zeiss Axio Imager M2 microscope equipped with an AxioCam HR Rev3 camera (Zeiss, Oberkochen, Germany). Images were processed using ImageJ software.

## 3 Results

### 3.1 Extraction efficiency: thermolysin vs. dispase

Epithelial cells and fibroblasts were extracted after the basal lamina was digested using either thermolysin or dispase. After initial enzymatic digestion, the epithelium can be mechanically separated from the stroma using tweezers.

Following tissue delamination, epithelial cells were isolated using trypsin and fibroblasts were isolated by a subsequent digestion with collagenase.

#### 3.1.1 Morphological observations

**FIGURE 2:**
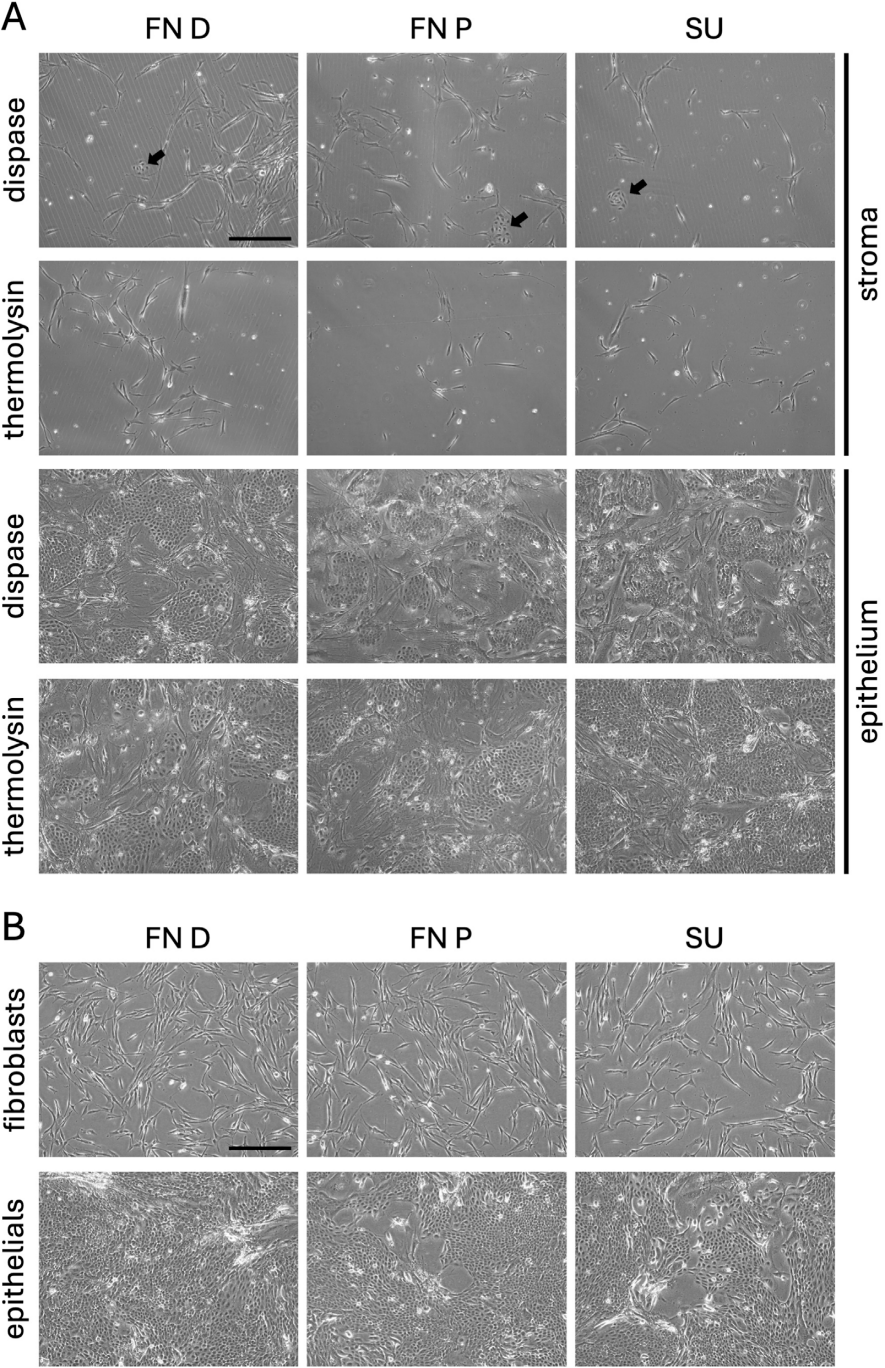
Morphology of isolated cell cultures. Cultured cells from the distal (left) or proximal (centre) Fossa Navicularis (FN D/FN P) or the spongy urethra (right, SU) are shown 7 days after enzymatic digestion **(A)** extracted by dispase II or thermolysin digestion. Morphology at passage 2 **(B)** is shown of cells extracted with dispase II at 2 days after seeding fibroblasts and 6 days for epithelial cells. Stromal cells and epithelial cells on a feeder layer are shown in bright field images. Scale bars represent 200 µm. Black arrows point to endothelial cell colonies found in the stromal cell culture.

Both fibroblasts and epithelial cells displayed typical morphology of the respective cell types using either thermolysin or dispase (Figure 2A). Fibroblasts showed a spindle-like, branched morphology. Interestingly, more endothelial cells were observed in stromal cultures when dispase was used; however, endothelial colonies were overtaken by fibroblasts as cultures reached confluence. Epithelial cells displayed a typical polygonal morphology with minimal intercellular spacing, suggesting tight cell-cell junctions. The cells grew in grapevine-like clusters. Colonies appeared to displace feeder cells as they grew. Epithelial cells began doubling immediately after seeding, in contrast to the fibroblasts, for which cultures appeared to stagnate before significant cell proliferation was observed.

Morphology was maintained throughout passages (Figure 2B). Subsequent passages grew in a more homogeneous manner, with fibroblasts evenly distributing on the culture surface, and epithelial cells growing in evenly spaced colonies, still displacing feeder cells as they reached confluence.

#### 3.1.2 Yield and stem cell maintenance over passages

**FIGURE 3:**
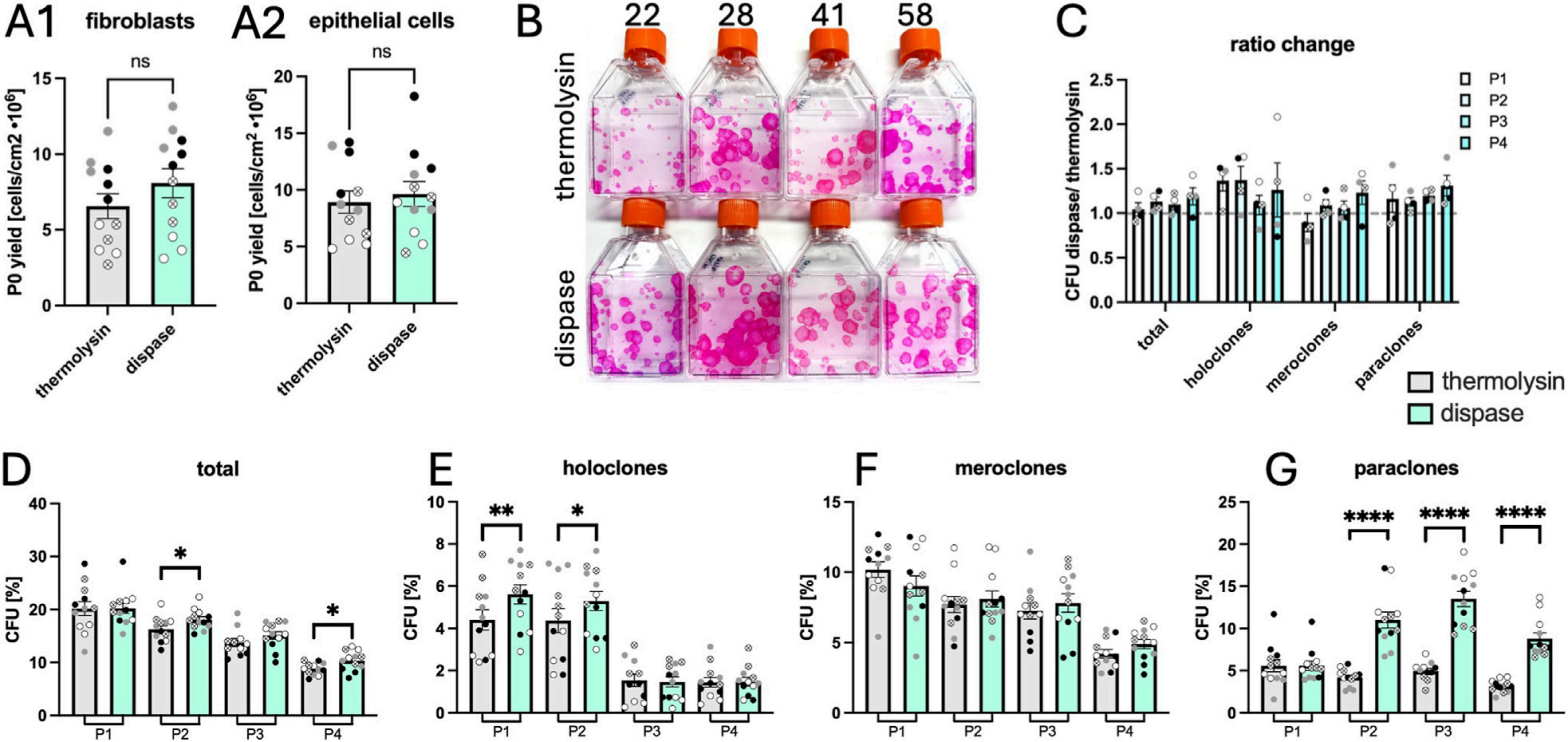
Yield and clonogenicity of isolated cells over 4 passages. Fibroblast **(A1)** and epithelial cell **(A2)** yield using thermolysin or dispase for tissue delamination. Representative colony staining using rhodamine is shown in **(B)** for passage 1. Patient age is indicated above. The colony-forming unit (CFU) ratios of dispase/thermolysin are shown in **(C)**, where a ratio above 1 indicates increased CFU in the dispase condition. CFU percentages are shown for total colonies **(D)**, holoclones **(E)**, meroclones **(F)**, and paraclones **(G)**. Symbols 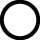, 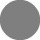, 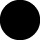, 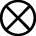 represent 22-, 28-, 41-, and 58-year-old donors. N = 12 (4 donors, 3 region-paired biopsies per donor). Statistical differences were calculated using ratio paired t-tests. Statistical significance is indicated as follows: p > 0.05 (ns), p < 0.05 (*), p < 0.01 (**), and p < 0.0001 (****). Exact p-values are given in Supplementary Table S1.

At the first passage after initial seeding (P0), cells were counted, and there was no significant difference between thermolysin or dispase conditions for yield of either fibroblasts (p = 0.0831, Figure 3A1) or epithelial cells (p = 0.4574, Figure 3A2).

To quantify population stemness, colony-forming units (CFU) were counted at passages 1–4 for both extraction protocols for epithelial cells. Colonies were larger for dispase conditions (Figure 3B). Colonies were classified according to diameter to identify holoclones (high stemness), meroclones (medium stemness), and paraclones (some stemness). CFU ratios for dispase over thermolysin protocols were calculated, and a ratio above 1 was always found for total clones, holoclones, and paraclones, even if statistical significance was not always determined. This indicates an increased efficiency of dispase in extracting stem cells. When evaluating increase in stem cell yields using dispase, for total CFU, a significant increase was only found for passages 2 and 4 (Figure 3D). However, when evaluating clone types individually, significantly more holoclones were present at passages 1 and 2 (Figure 3E). Significantly more paraclones were identified from passages 2 to 4 (Figure 3G), although paraclone presence was most likely masked at passage 1 due to large colony size. Total CFU decreased slightly at each passage, similarly to meroclones; however, holoclone content dropped for both protocols 3-fold between passages 2 and 3. Overall, epithelial populations are seen to decrease both in quantity and quality of stem cells over passages, with dispase-extracted populations maintaining superior quality over passages. In Supplementary Figure S1, more SOX17 positive cells were observed in the dispase condition compared to thermolysin (17.5% and 21.9%, respectively, p = 0.063) supporting that dispase preserves more stem cells.

### 3.2 Stromal extraction optimizations

Following dispase digestion and removal of the epithelium, stromal compartments were digested for 4 or 20 h with collagenase ± elastase to maximize fibroblast yield.

**FIGURE 4:**
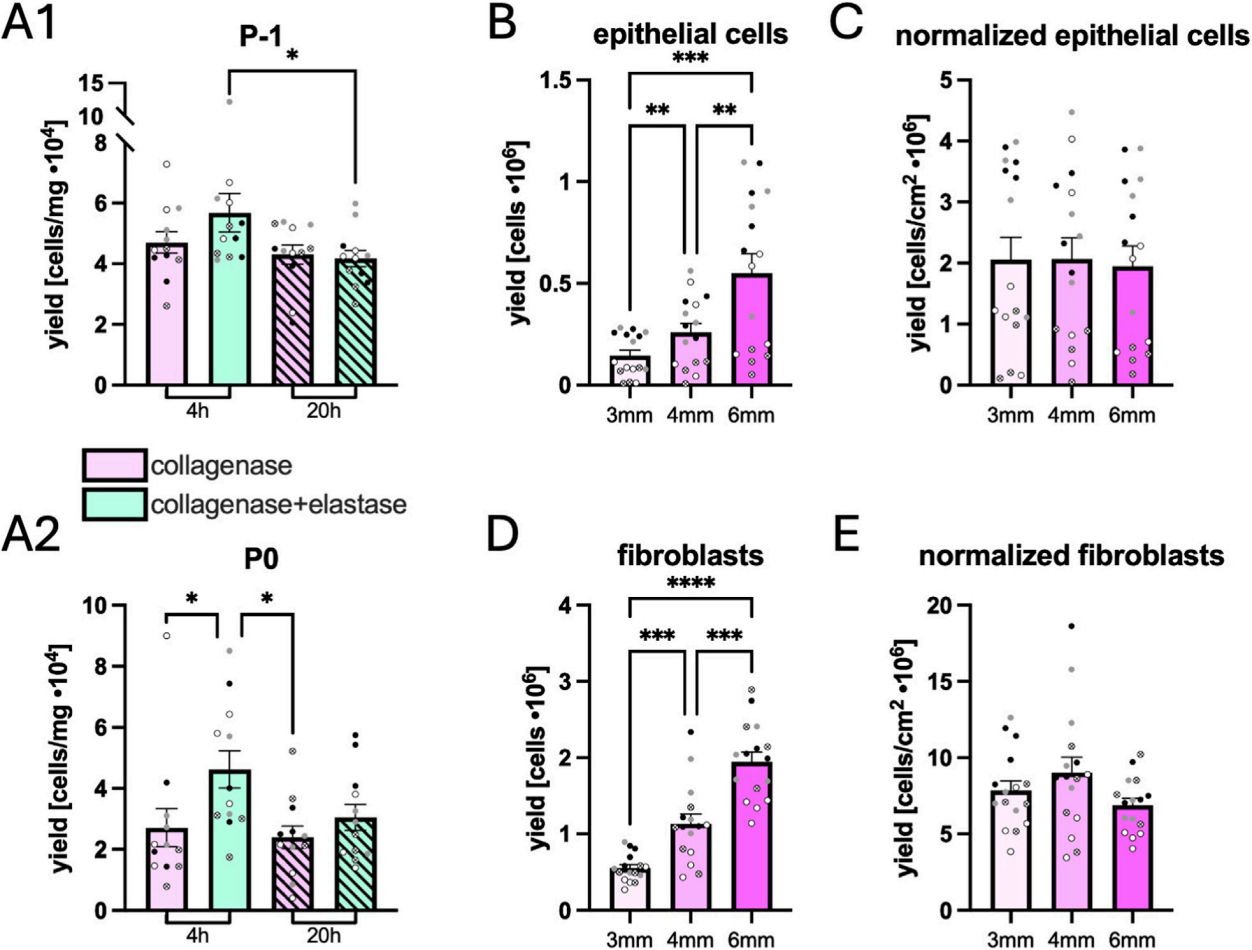
Influence of elastase and biopsy punch size on cell extraction yields. Stromal cell yields with or without elastase and incubated 4 or 20 h **(A)**. Yields were quantified using a Coulter Z2 counter immediately after digestion, before first seeding (P-1) **(A1)**, and at first passage (P0) **(A2)**. Symbols 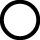, 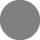, 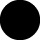, 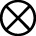 represent 19-, 35-, 39-, and 52-year-old donors. N = 4 donors, n = 3. Statistical differences were evaluated using a 2-way ANOVA followed by a Tukey’s test. Effect of biopsy punch diameter on cell extraction yield **(B–E)**. Epithelial cells **(B,C)** and fibroblasts **(D,E)** were quantified immediately after enzymatic digestion (P-1). Both total yields **(B,D)** and surface normalized yields **(C,E)** are represented. N = 16 (4 donors, 4 region-paired biopsies). Statistical differences were evaluated using a 1-way ANOVA followed by a Tukey’s test. Asterisks indicate statistical significance, p < 0.05 (*), p < 0.01 (**), p < 0.001 (***) and p < 0.0001 (****). Exact p-values are given in Supplementary Tables S2 and S3.

Cells were counted before initial seeding (P-1) and at P0. A significant difference was only found for P-1 between elastase conditions at 4 and 20 h (Figure 4A). At P0, 70% more cells were collected when adding elastase for 4 h during incubation, but 20 h of incubation did not significantly negatively affect cell yield.

### 3.3 Determining minimum punch diameter for tissue biopsy

To understand the effect of biopsy punch size and to determine the minimum size necessary for cell extraction, biopsy punches of 3, 4, and 6 mm were used to standardize tissue surface areas. Tissues were delaminated using dispase, and fibroblasts were isolated with collagenase and elastase. Cells were counted at P-1 (Figures 4B–E). When cell counts were normalized by the surface area, no significant difference was found in the yields, indicating little to no border effects were observed and that even the smallest of biopsies were manipulable.

### 3.4 Immunofluorescence characterization

#### 3.4.1 Fibroblasts

**FIGURE 5:**
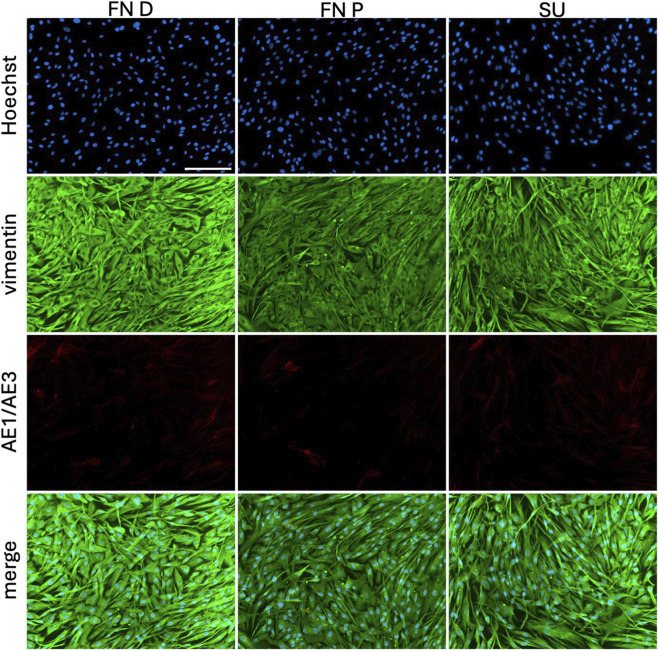
Purity of extracted fibroblasts. From the distal or proximal fossa navicularis (FN D, FN P) or the spongy urethra (SU) using dispase, followed by collagenase with elastase. Immunofluorescence images of fibroblasts (P3) at confluence stained with Hoechst (blue), vimentin (green), and AE1/AE3 (red). Scale bar represents 200 µm.

Fibroblasts at passage 2 were immunolabeled at confluence for vimentin and AE1/AE3 (Figure 5). All cells exhibited strong filamentous staining for vimentin, consistent with fibroblast identity. In contrast, AE1/AE3 staining was weak and limited, with no cells showing strong or filamentous cytokeratin expression. The cells displayed a uniform spindle-shaped morphology typical of fibroblasts.

#### 3.4.2 Epithelial cells

A large panel of antibodies was used to immunolabel the isolated epithelial cells. These cultures were compared to previously characterized vaginal and bladder epithelial cell populations, serving as references for cells of similar embryological origin or urothelial lineage, respectively.

**FIGURE 6:**
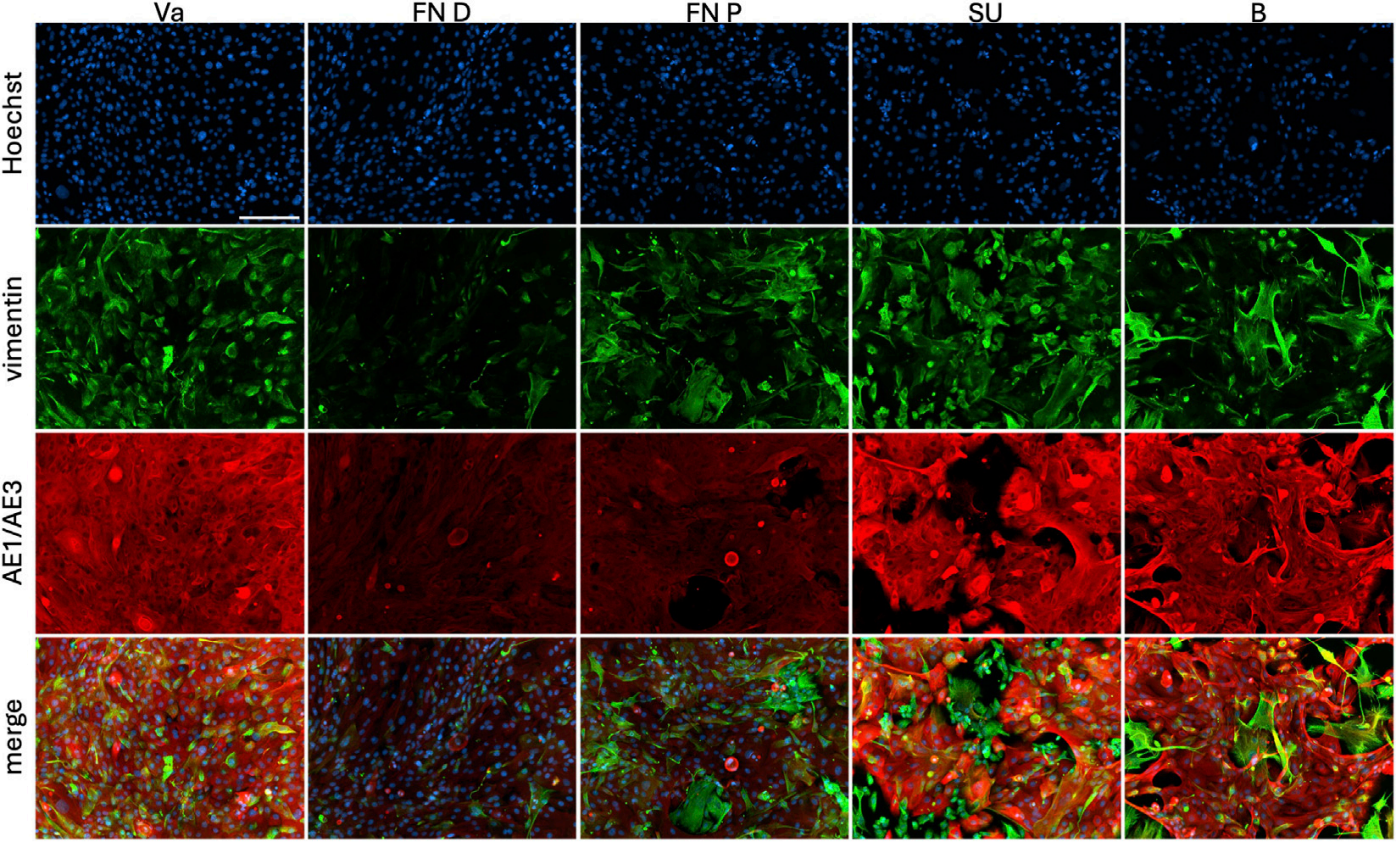
Characterization of extracted epithelial cells using dispase II. From the distal or proximal fossa navicularis (FN D, FN P) or the spongy urethra (SU), cells were compared to vaginal epithelial cells (Va) and bladder epithelial cells (B). Cells were stained with Hoechst (blue), vimentin (green) and pan-cytokeratin (AE1/AE3, red). Presence of vimentin in epithelial culture resulted from the use of a feeder-layer (irradiated human foreskin fibroblasts). Scale bar represents 200 µm.

Co-labelling for vimentin and AE1/AE3 was performed (Figure 6). All populations exhibited filamentous vimentin staining in a subpopulation attributable to the human dermal fibroblast feeder cells, and AE1/AE3 labelling was present in all epithelial cells. A small subset of cells in each population showed co-localization of both markers, and it was noticed that AE1/AE3 labelling was weaker in both FN populations.

**FIGURE 7:**
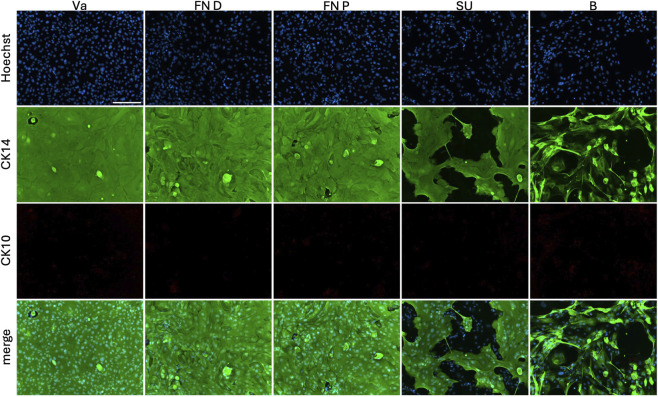
Characterization of extracted epithelial cells using dispase II. From the distal or proximal fossa navicularis (FN D, FN P) or the spongy urethra (SU), cells were compared to vaginal epithelial cells (Va) and bladder epithelial cells (B). Cells were stained with Hoechst (blue), cytokeratin 14 (CK14, green) and cytokeratin 10 (CK10, red). Scale bar represents 200 µm.

CK14 and CK10 were co-labeled in the different epithelial populations (Figure 7). CK14 is characteristic of all layers of stratified squamous epithelia, while CK10 typically marks the differentiated layers of vaginal and FN epithelia. In vaginal and both FN populations, a confluent layer of CK14 staining was observed. CK14 labelling highlighted cell borders encompassing multiple nuclei, indicating a multilayered organization in these cultures. This was confirmed by confocal imaging (Supplementary Figure S2). In contrast, epithelial cells from the spongy urethra (SU) population did not exhibit homogenous CK14 labelling. Bladder epithelial cells were positive for CK14; however, the staining was less homogeneous, and no large, flattened cells covering multiple nuclei, as seen in the other populations, were observed. CK10 was absent from all populations, consistent with limited differentiation. Antibody specificity was verified with native vaginal tissue (Supplementary Figure S3).

**FIGURE 8:**
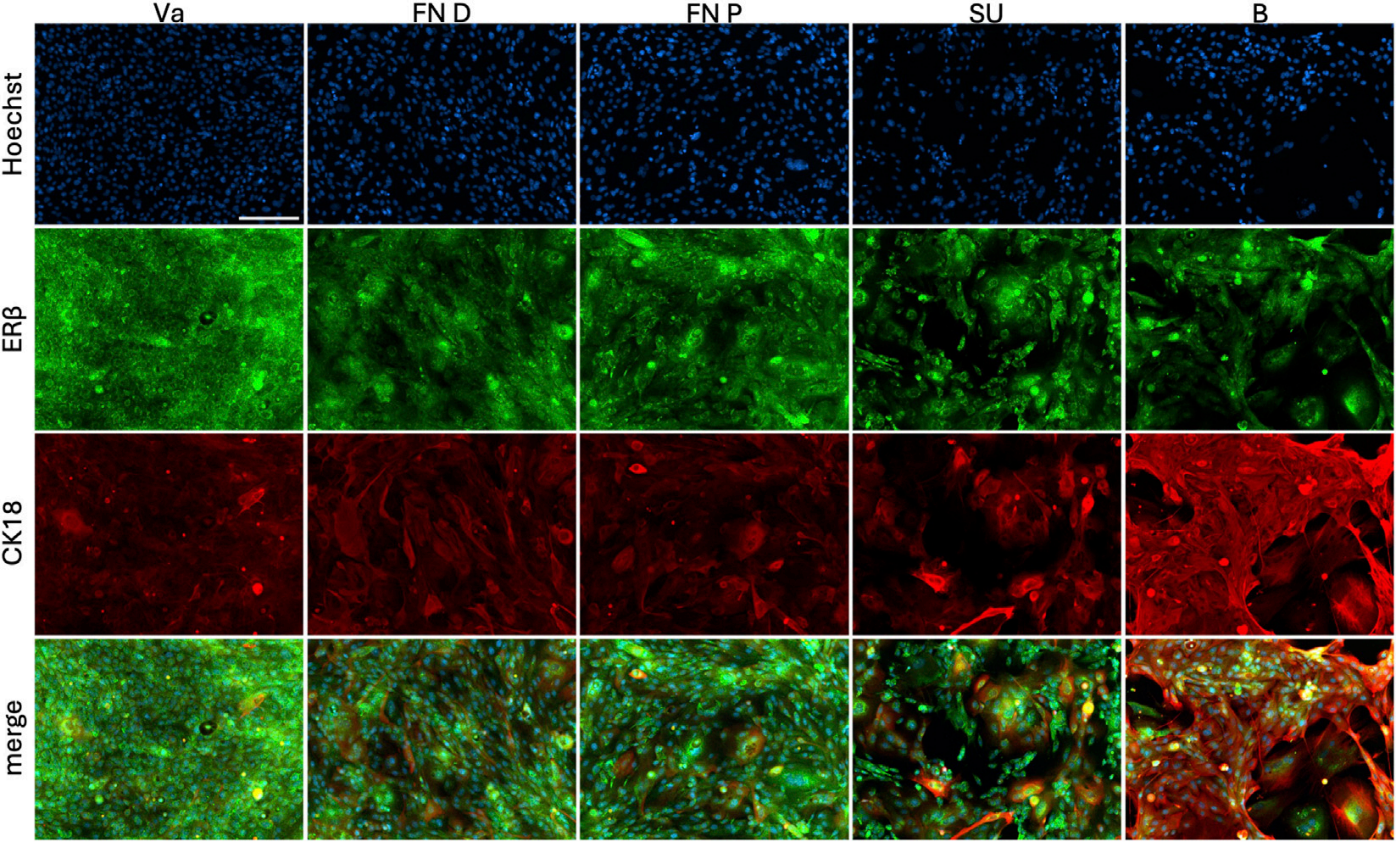
Characterization of extracted epithelial cells using dispase II. From the distal or proximal fossa navicularis (FN D, FN P) or the spongy urethra (SU), cells were compared to vaginal epithelial cells (Va) and bladder epithelial cells (B). Cells were stained with Hoechst (blue), estrogen receptor beta (ERβ, green) and cytokeratin 18 (CK18, red). Scale bar represents 200 µm.

Estrogen receptor beta (Erβ) and CK18 were co-labelled in the epithelial populations (Figure 8). All cell populations exhibited granular ERβ staining, predominantly cytoplasmic with occasional focal nuclear localization. Vaginal and urethral epithelial cells showed strong perinuclear ERβ labeling, whereas bladder epithelial cells displayed a more diffuse, less perinuclear pattern.

CK18 expression was detected in a scattered subpopulation of vaginal and urethral epithelial cells. In the SU population, CK18 staining was more intense compared to the FN populations. In contrast, all bladder epithelial cells expressed CK18 uniformly.

**FIGURE 9:**
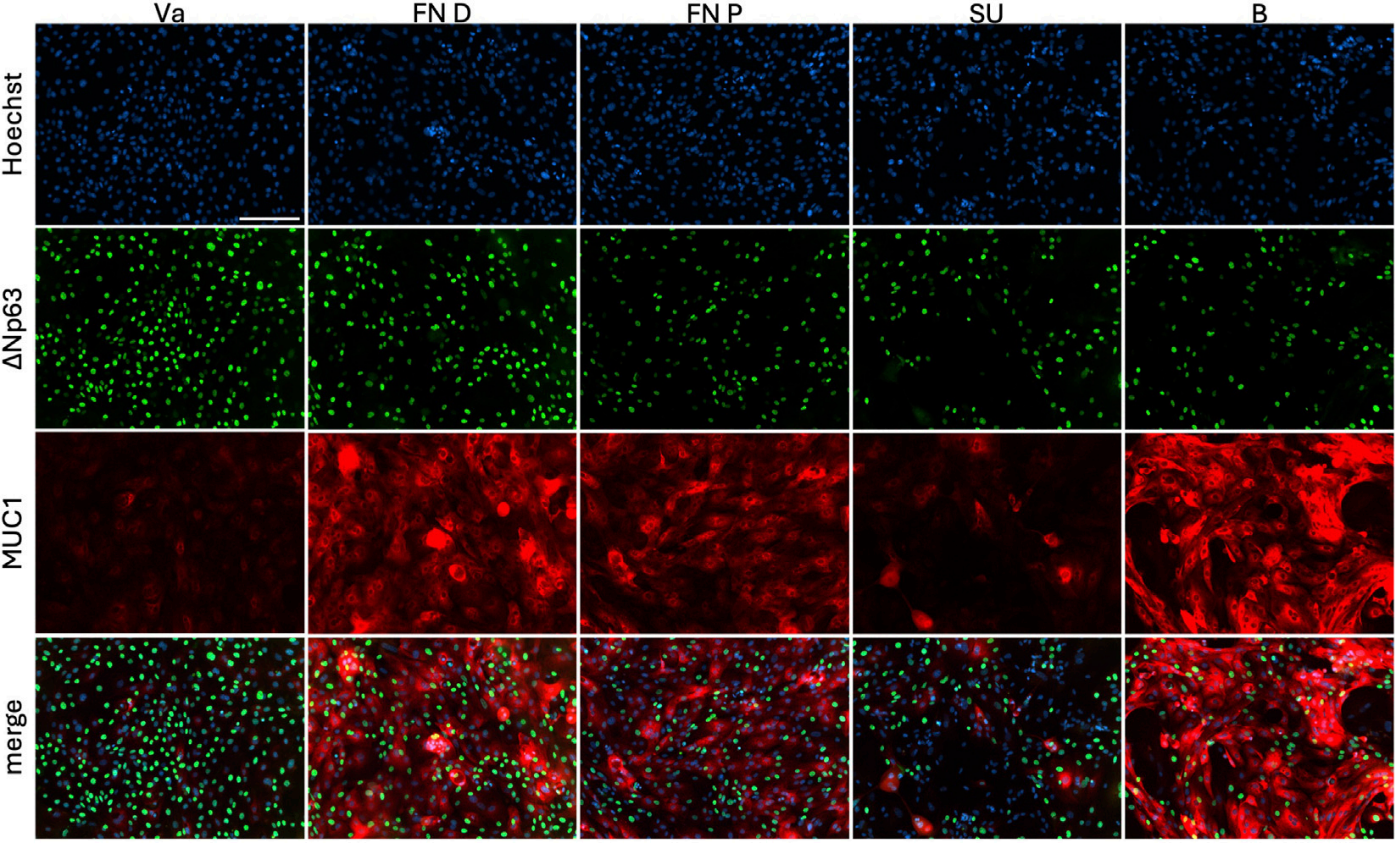
Characterization of extracted epithelial cells using dispase II. From the distal or proximal fossa navicularis (FN D, FN P) or the spongy urethra (SU), cells were compared to vaginal epithelial cells (Va) and bladder epithelial cells (B). Cells were stained with Hoechst (blue), ΔNp63 (green) and mucin 1 (MUC1, red). Scale bar represents 200 µm.

ΔNp63 and MUC1 were co-labeled in all epithelial populations (Figure 9). The percentage of ΔNp63-positive cells in the vaginal, distal FN, proximal FN, SU, and bladder populations was 55%, 40%, 29%, 21%, and 33%, respectively. MUC1 expression was detected in all populations, with stronger staining observed in the FN and bladder-derived cells. ΔNp63 and MUC1 did not co-localize, suggesting that mucus production is restricted to more differentiated cells within the cultures.

## 4 Discussion

In this study, the first successful protocol for the extraction and subculture of epithelial cells and fibroblasts from the human male urethral mucosa was established. Cells were reliably isolated from all three anatomically distinct regions of the urethra, with a 100% success rate across all evaluated protocols. The extracted epithelial cells maintained progenitor characteristics over multiple passages *in vitro*, underscoring their potential for tissue engineering applications. Importantly, these protocols provide a robust source of autologous urethral epithelial cells and fibroblasts, which may support future efforts for *in vitro* tissue reconstruction and personalized disease modelling.

Although the findings of this study demonstrate that dispase-based extraction preserves epithelial populations with strong progenitor-like characteristics, the methods employed cannot definitively establish the presence of urethral stem cells or confirm their long-term self-renewal and differentiation potential. Morphology, colony-forming efficiency, and ΔNp63 expression are standard and widely accepted indicators of progenitor preservation in epithelial tissues such as skin; however, these features have not yet been validated as definitive markers of stemness in the penile urethra. As this is the first study to report the extraction and characterization of human penile urethral epithelial cells, rigorous identification and characterization of urethral stem cells would require additional approaches such as serial clonogenic passaging, long-term assays evaluating differentiation capacity, and assessment of an expanded panel of stem cell markers, which fall outside the scope of the present work. The primary aim of this study was to compare extraction protocols and optimize cell yield and performance for tissue engineering applications. Accordingly, while the results support the retention of progenitor-enriched populations, definitive conclusions about stem cell identity, self-renewal, or differentiation potential cannot be drawn from the current methods.

Organ-specific tissue reconstruction is critical when selecting cell sources for tissue engineering (Culenova et al., n.d.; Žiaran et al., 2017; Caneparo et al., 2022; Yuan et al., 2025). Many current urethral tissue engineering approaches rely on nonhuman cells (De Graaf et al., 2024; Casademont-Roca et al., 2025) or bladder-derived urothelium (Guan et al., 2008; Cattan et al., 2011; Raya-Rivera et al., 2011; Fossum et al., 2012; Orabi et al., 2013; Amesty et al., 2021; Caneparo et al., 2022), largely due to a historical gap in knowledge surrounding the histology and cellular identity of the penile urethra. While bladder urothelium may offer effective barrier function and protect against urine leakage, thus mitigating cytotoxic injury to surrounding tissues, it likely originates from a distinct cell lineage and does not recapitulate the region-specific physiological roles of the native urethral mucosa (Wu et al., 2009).

The urethra is not a uniform tube, but a series of specialized epithelia (Duval et al., 2025), each shaped by its developmental origin and physiological role. Tissue engineering grafts should reflect this complexity. Anatomically matched mucosal cells may offer better functional outcomes, enhanced tissue integration, and more stable long-term regeneration. For example, in cases of minor hypospadias, distal FN-derived epithelium may be sufficient for repair. Conversely, for strictures involving the SU, SU-specific epithelial cells would be more appropriate due to their urothelial phenotype. In complex cases such as severe hypospadias, it may be conceivable to engineer a zonal graft incorporating multiple cell types to mimic native urethral architecture.

Beyond mechanical and immunological compatibility, region-specific epithelia may also help restore the native microbiome. FN D epithelium, for instance, shares histological and embryological similarities with vaginal mucosa and is believed to support colonization by lactobacilli, which contribute to acidification of the mucosal environment and defense against pathogens (Nelson et al., 2012).

Once the biopsy area is selected, the first step in cell extraction from bilaminar tissues via enzymatic digestion is the separation of the epithelium from the stroma (Mirbolooki et al., 2011). These two layers are firmly connected through the basement membrane, which must be digested to allow effective delamination and recovery of pure epithelial and stromal populations. Two commonly used neutral proteases were evaluated for this step: thermolysin and dispase II (Ścieżyńska et al., 2019).

First, thermolysin is a thermostable zinc-metalloproteinase. It cleaves peptide bonds on the N-terminal side of hydrophobic amino acids, allowing efficient digestion of extracellular matrix (ECM) components within the basal lamina while sparing most epithelial surface proteins. Its thermostability, conferred by calcium binding, adds to its robustness in tissue dissociation protocols. While thermolysin exhibits broad substrate specificity, it is non-collagenolytic and may affect certain cell surface proteins, including those on the plasma membrane, which could influence epithelial cell viability and stemness (Lockhart and S. Hakakian, 2015).

Second, dispase II is widely used for the gentle dissociation of epithelial tissues. It selectively cleaves ECM proteins such as fibronectin, collagen IV, and to a lesser extent collagen I, while mostly sparing collagen V and laminin, key components that help preserve epithelial architecture (Lockhart and S. Hakakian, 2015). Activated by calcium ions, dispase II acts primarily in the basement membrane zone, enabling the detachment of intact epithelial sheets while maintaining intercellular junctions and cell surface markers. Unlike thermolysin, which degrade a wide range of ECM proteins indiscriminately, dispase II acts more selectively, minimizing damage to the epithelial cell surface and niche. This gentler enzymatic activity is particularly advantageous for preserving the integrity and function of basal progenitor cells during extraction (Rakhorst et al., 2006).

As such, it was hypothesized that dispase II would yield an epithelial population enriched in progenitor cells. This was confirmed through CFU assays over four passages, where dispase II-extracted epithelial cells consistently outperformed those extracted using thermolysin. They formed more numerous and larger colonies, likely reflecting the gentler, more selective action of dispase II. This is particularly important given that enzymatic cleavage occurs directly adjacent to the basal layer of the epithelium, where epithelial stem and progenitor cells are concentrated. Excessive or non-specific proteolysis in this region could lead to premature differentiation or loss of proliferative capacity (Lu et al., 2010).

These findings are consistent with previous literature. A study by Matsui et al. [29] showed that dispase II digestion resulted in a higher epithelial yield than thermolysin in rabbit oral mucosa (Rakhorst et al., 2006). Similarly, a methods review by Lynch et al. concluded that dispase is the optimal enzyme for keratinocyte isolation due to its ability to preserve cell-cell junctions and stem-like characteristics (Ścieżyńska et al., 2019).

The mucosal ECM is a complex network of structural proteins that supports tissue architecture and regulates cell behaviour (Manou et al., 2019). In the urethral stroma, collagen I is the predominant ECM component, providing tensile strength. However, due to the mucosal nature of the urethra, elastin also plays a key role in contributing elasticity and compliance, essential for withstanding dynamic changes during urination and sexual activity (Bastos et al., 2004).

Compared to the explant method, enzymatic digestion enables significantly greater and more rapid fibroblast yield from biopsy samples (Wang et al., 2004). Moreover, explanting cells induces a strong bias for migratory cells, which is avoided using enzymatic digestion (Mushahary et al., 2018). While it is often assumed that fibroblast yield increases proportionally with the extent of ECM digestion, this relationship is not strictly linear. As matrix components are digested, fibroblasts may remain adherent to partially degraded ECM fragments rather than being fully released into suspension (Solomonov et al., 2016). Consequently, during the filtration step, fibroblasts still bound to ECM remnants may be retained on the filter, leading to an underestimation of yield. This underscores the importance of optimizing enzymatic digestion not only to degrade matrix proteins but also to ensure efficient cell release into the suspension phase.

Although one may consider omitting the filtration step to avoid such loss, ECM debris remaining in culture often floats in the medium with little cell attachment. Moreover, given the highly vascular nature of the urethral mucosa, skipping the filtration step increases the risk of endothelial contamination, reducing fibroblast purity (Normand and Karasek, 1995).

Fibroblast yield was optimized by testing different enzymatic digestion conditions. The highest yield was obtained using a 4-h digestion with collagenase I/II and elastase, while other enzyme conditions did not significantly improve yield. Although it might be expected that prolonged digestion (20 h) would result in more complete ECM degradation and greater cell release, the lack of improvement suggests that cytotoxic effects may offset this benefit. Elastase, in particular, may exert greater cytotoxicity over extended incubation, potentially explaining the reduced effectiveness of the combined enzyme condition at 20 h. Cell death could also have been due to anoikis induced by prolonged duration of cell suspension which may be improved by the use of ROCK inhibitor Y-27632 (Zhao et al., 2019).

It is important to interpret the yield data at P-1 with caution, as these cells had not yet been cultured and their viability was unknown. Although no statistically significant difference was found between groups, the actual number of viable fibroblasts may have varied. Interestingly, similar cell counts were obtained at P-1 and P0, indicating the naturally low survival rate of extracted cells. This highlights the necessity of high seeding density: low initial fibroblast density is known to result in delayed proliferation due to insufficient cell-cell signalling and reduced attachment efficiency.

Importantly, the results suggest that if time constraints prevent immediate processing, stromal sections can be digested overnight with collagenase I/II alone without compromising final yield. It must be noted that all conditions were assessed at the same time point after initial seeding. This means the 4-h condition benefited from an additional 16 h of culture time before cell counting at P0, which may partially explain its apparent advantage. Had cultures been assessed at confluence rather than fixed timepoints, the observed differences in yield might have diminished. Therefore, the main benefit of fibroblast extraction optimization lies not only in maximizing cell numbers but also in accelerating culture timelines, a key consideration in tissue engineering workflows, particularly when preparing autologous grafts for clinical use.

When extracting cells from patient biopsies for autologous tissue engineering, it is critical to minimize patient morbidity, particularly when sampling from sensitive regions such as the urethra. Unlike skin biopsies, where several square centimetres can be harvested without major impact, removing even small areas of urethral mucosa poses greater clinical risks and recovery challenges. This is especially relevant given that patients must live with the biopsy wound during the *in vitro* culture period, which can extend over several weeks to months. Therefore, to assess the feasibility of minimizing biopsy size, epithelial and stromal cells were isolated from tissue samples obtained using 3, 4, and 6 mm biopsy punches, corresponding to surface areas of approximately 7.1 mm^2^, 12.6 mm^2^, and 28.3 mm^2^. Cells were seeded in 1, 2, or 4 wells of a 6-well plate, respectively, yielding approximately 1 × 10^6^ cells per well at P0, suggesting that even the smallest biopsies can generate sufficient cells for downstream applications.

When considering use in tissue engineering, particularly for urethral reconstruction, the required biopsy size can be retrospectively calculated from the cell density requirements of specific fabrication methods. For example, using the self-assembly tissue engineering technique, typical epithelial seeding densities range from 1.7 × 10^5^ to 6.7 × 10^5^ cells/cm^2^. For an entire penile urethra of 15 cm in length and 1 cm in diameter, the total surface area to reconstruct is approximately 47 cm^2^, requiring between 8.0 × 10^6^ and 3.1 × 10^7^ epithelial cells.

Given that epithelial stemness declines notably after P3, the most accurate estimation should be based on yields at P2. The results suggest that a 3 mm biopsy punch can yield over 1 × 10^8^ epithelial cells by P2, indicating that such a minimally invasive biopsy is likely sufficient for full urethral reconstruction in adults. For pediatric patients or smaller grafts, even smaller biopsies could be considered.

Additionally, strategies such as hypoxic culture conditions could help maintain epithelial progenitor potential over extended passages, potentially further reducing the initial biopsy size required (Bath et al., 2013).

Immunofluorescence labelling was used to confirm the identity and purity of epithelial and stromal cell populations extracted from distinct anatomical regions of the urethral mucosa. AE1/AE3 and vimentin co-labelling supported the purity of the stromal isolations. In epithelial cultures, vimentin-positive cells were observed, but the morphology was consistent with the phenotype of the irradiated dermal fibroblast feeder cells used to support epithelial growth. The absence of fibroblast outgrowth in any of the epithelial cultures further supports the specificity and purity of the extraction protocols.

While feeder-free systems are under development (Yu et al., 2015), the use of irradiated human dermal fibroblast feeders remains a widely accepted, cost-effective method to preserve stemness in primary epithelial cultures (Bisson et al., 2013). These cells are non-proliferative, gradually die off, and have been used clinically for decades without reported complications (Llames et al., 2015). Moreover, human fibroblasts are known to have low immunogenicity (Deshpande et al., 2010; Magne et al., 2024), reducing concerns about potential rejection in future allogeneic applications.

Further immunophenotyping focused on epithelial populations, as few specific markers are known for urethral fibroblasts. To assess regional epithelial identity, epithelial cells derived from vaginal mucosa and bladder urothelium were used as controls. FN epithelium is of the same embryological origin as the vaginal epithelium (Baskin et al., 2018) and displays similar histological features, while SU is histologically classified as a urothelium (Duval et al., 2025).

Despite similar seeding densities, SU-derived epithelial cultures did not form a confluent monolayer, which may reflect a urothelial phenotype. Similar findings have been reported for porcine urethral urothelial cells requiring dynamic flow conditions to achieve confluence (Casademont-Roca et al., 2025). CK14 expression was observed in FN D, FN P, and vaginal cells, consistent with a stratified squamous phenotype. In contrast, CK10 was absent in all cultures, suggesting limited terminal differentiation, as this marker is typically restricted to superficial layers of squamous epithelia.

ERβ was expressed in all epithelial populations. While this is expected in vaginal cells, its presence in urethral epithelium is less well documented but consistent with reports of hormonal responsiveness in the lower urinary tract (Dietrich et al., 2004). CK18, typically associated with simple or urothelial epithelia, was detected in all bladder controls and in a large subpopulation of vaginal and urethral epithelial cells. As CK18 is not characteristic of vaginal epithelium, its presence in cultures may reflect a degree of phenotypic plasticity *in vitro* or retention of transitional epithelial features as previously described by Fichorova et al. (1997).

All epithelial populations displayed significant subpopulations of ΔNp63-positive cells, confirming the clonogenic potential observed in CFU assays. Expression was more prominent in the vaginal, FN D, and FN P populations, in keeping with the known enrichment of the ΔN isoform of p63 in stratified squamous epithelia (Medawar et al., 2008) compared to urothelia. Co-labelling with MUC1 showed positive staining in all populations, consistent with their mucosal origin. As expected, MUC1 and ΔNp63 did not co-localize, reflecting the spatial separation of basal progenitor and apical differentiated compartments within the epithelial hierarchy.

Overall, these results confirm the epithelial identity and purity of the isolated populations and highlight region-specific differences in marker expression. The similarity between FN-derived and vaginal epithelial populations reinforces their developmental and functional relatedness and supports their use in mucosal tissue engineering. Further characterization of the SU epithelium is warranted to better define its phenotype, particularly given its potential transitional characteristics.

Given the striking similarity between FN D and vaginal epithelia, FN D cells may also represent a promising autologous source for vaginal reconstruction in male-to-female transgender patients. Currently, skin grafts, which lack mucosal characteristics, are commonly used to line the neovaginal cavity (Pariser and Kim, 2019). Grafting with FN D-derived cells could offer a more physiologically appropriate mucosal surface, potentially providing self-lubrication and enhanced barrier properties critical for comfort, sexual function, and microbial homeostasis. These applications warrant further investigation through preclinical models and translational research.

Beyond its applications in reconstructive tissue engineering, the ability to isolate region-specific urethral epithelial and stromal cells offers a valuable platform for *in vitro* disease modelling. Despite the high prevalence of congenital and acquired urethral disorders such as hypospadias and strictures, there are currently no established human *in vitro* models to study these conditions at the cellular level (de Kemp et al., 2015; Casademont-Roca et al., 2025). This gap has limited mechanistic understanding and hindered the development of targeted therapeutic strategies.

Epithelial cell extraction from mucosal tissues is notoriously challenging because of the fragile epithelial-stromal interface. Our protocol provides improved reproducibility and flexibility, allowing overnight Dispase II incubation and adjustable collagenase digestion (4–20 h) without compromising cell viability or progenitor preservation. This flexibility facilitates clinical sample processing while maintaining epithelial quality. Notably, previous comparative studies on mucosal cell isolation, such as the recent work by Sueters et al. (2025), failed to establish viable epithelial cultures despite testing multiple enzymatic combinations. To our knowledge, this study represents the first quantitative comparison of enzymatic extraction strategies for the human urethra, thereby filling an important methodological gap in mucosal tissue isolation.

The extraction method presented here enables consistent recovery of viable, region-specific epithelial and stromal cells and has also been successfully applied to other mucosal tissues, bladder and vagina. Based on this versatility, the protocol should be readily adaptable to diseased urethral specimens, enabling the development of biologically relevant models.

These cells can serve as a foundation for a range of experimental systems, from 2D monolayer cultures to 3D organoid models to tissue-engineered constructs, depending on the complexity required. Such platforms could be used to explore hormonal, inflammatory, or ECM-related mechanisms underlying urethral pathology or to evaluate responses to candidate therapies under defined and reproducible conditions. By making these cell types more experimentally accessible, this work supports the development of urethra-specific disease models that are both biologically relevant and technically feasible across a range of experimental formats.

Despite the significant advances presented in this study and its contributions to urethral research, several limitations must be acknowledged. Although cells from 12 donors were successfully isolated, only a limited number of populations were fully characterized. More comprehensive techniques, such as single-cell proteomics, could provide a more precise identification of the isolated cell populations and allow comparison to native, unextracted urethral cells.

Furthermore, cells were extracted from male-to-female transgender patients who had undergone hormone therapy. While this is advantageous for developing grafts tailored to this population, hormonal treatment may have influenced cell phenotype, especially given the presence of ERβ.

Finally, the use of 2D culture systems poses another limitation, as they often fail to replicate the complex *in vivo* environment. In particular, the use of dermal fibroblast feeder layers may have influenced epithelial cell behaviour and gene expression, potentially limiting the physiological relevance of the observed phenotypes.

These findings open several avenues for further investigation. The application of the extracted urethral cells in tissue engineering and *in vitro* modelling is a logical next step. Given the presence of progenitor-rich populations, it should be feasible to adapt established bilaminar reconstruction protocols such as those used for skin and vaginal tissue, to urethral contexts. This could support the development of patient-specific grafts and experimental platforms for disease modelling.

The detection of endothelial cells in primary fibroblast cultures also suggests that protocol refinements could allow for the selective expansion or isolation of urethral endothelial cells. Incorporating these cells into engineered tissues may increase model complexity and physiological relevance. In the longer term, efforts could be directed toward isolating additional cell types, such as resident immune or nerve cells, enabling the development of even more comprehensive urethral tissue models.

## 5 Conclusion

This study presents the first successful and reproducible protocol for isolating epithelial cells and fibroblasts from anatomically distinct regions of the human penile urethra. The optimized use of dispase II, collagenase, and elastase enabled high cell yields and the preservation of progenitor characteristics, even from minimal biopsy samples. These findings establish a robust foundation for autologous urethral tissue engineering and open new avenues for disease modelling using organ-specific human cells. By making these rare cell populations experimentally accessible, this work directly addresses long-standing limitations in the field and supports the development of anatomically and functionally relevant reconstructive and investigative platforms.

## Data Availability

The raw data supporting the conclusions of this article will be made available by the authors, without undue reservation.
