# Supplementary figures and images for: Isolation and characterization of epithelial cells and fibroblasts from the human penile urethra
Source: Front Bioeng Biotechnol. 2025 Dec 10;13:1713156. doi: 10.3389/fbioe.2025.1713156 (PMC12728052; doi:10.3389/fbioe.2025.1713156)

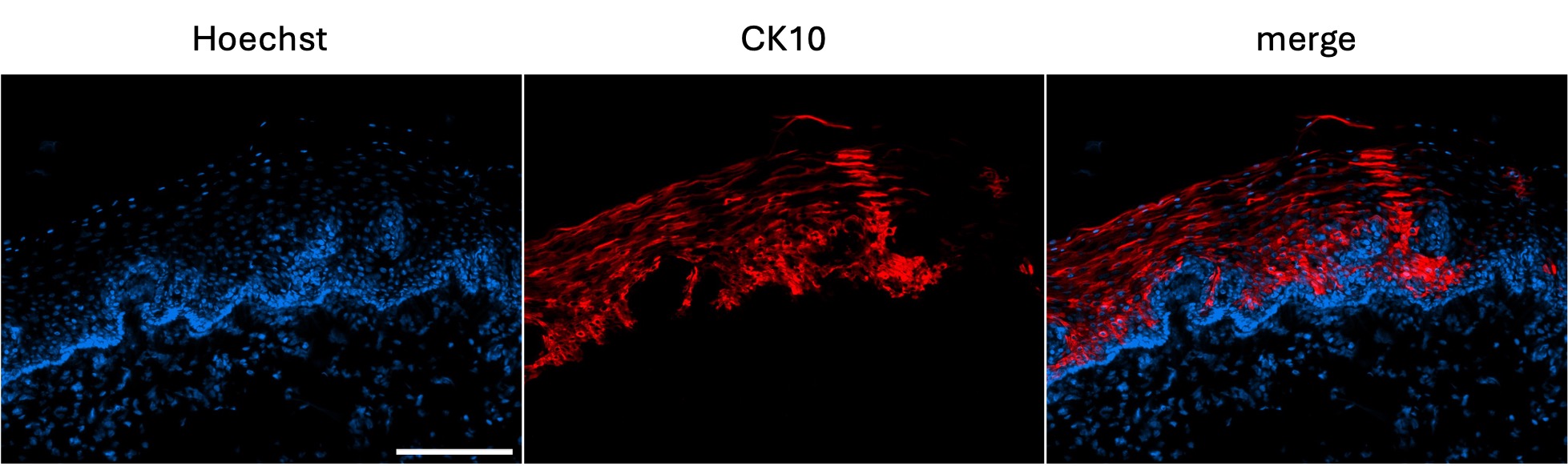

Supplement: Supplementary file 1 [file Image3.jpeg]

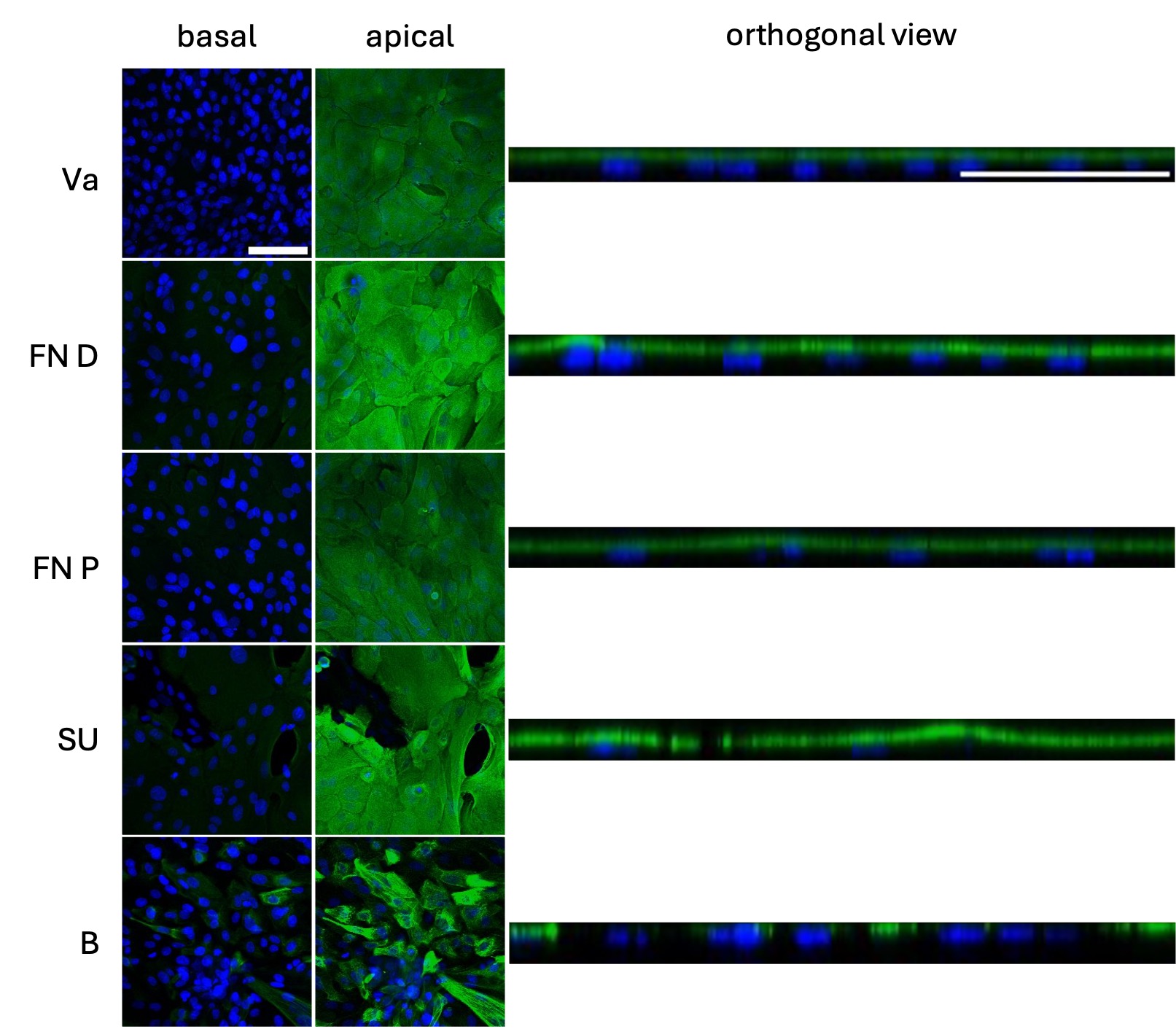

Supplement: Supplementary file 2 [file Image2.jpeg]

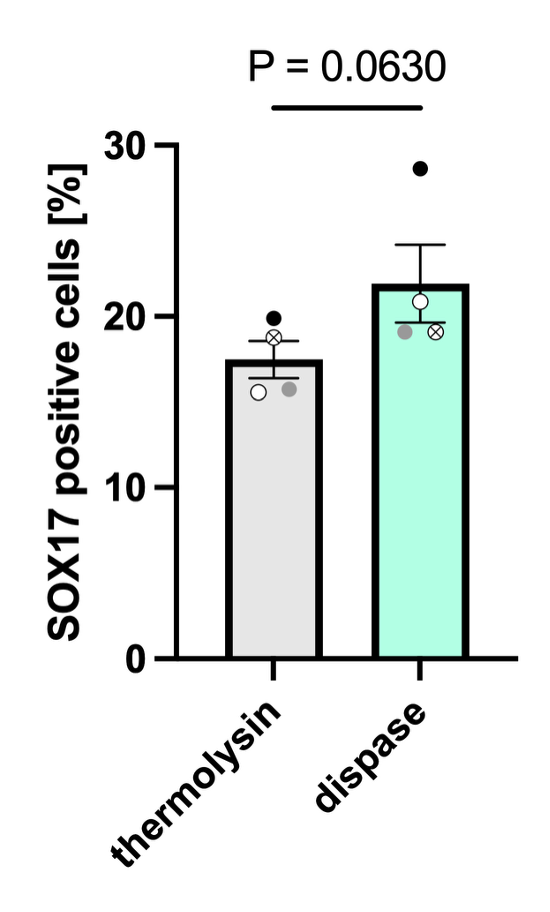

Supplement: Supplementary file 4 [file Image1.png]
