# Supplementary material for: Isolation and characterization of epithelial cells and fibroblasts from the human penile urethra
Source: Front Bioeng Biotechnol. 2025 Dec 10;13:1713156. doi: 10.3389/fbioe.2025.1713156 (PMC12728052; doi:10.3389/fbioe.2025.1713156)
Supplement: Supplementary file 3 [file DataSheet1.pdf]

Supplementary Methods

Flow cytometry

Briefly, 1 million epithelial cells at P1 from four thermolysin or dispase extracted populations (22-, 28-, 41-, or 58-year-old) were washed once with PBS before fixation in a HEPES buffered 4% PFA solution (4% paraformaldehyde, 137mM NaCl / 3mM KCl / 20mM HEPES / pH7.4). The PFA was inhibited with an equal volume FACS PBS buffer (PBS / 2% FBS / 0.5µM EDTA / pH 7.4) then cells were permeabilized in 100% methanol. The cells were washed in PBS containing 5% BSA, then in FACS PBS buffer before staining for SOX17 for 45 minutes with an AlexaFluor488 coupled antibody (BD Biosciences 562205). An isotype control (BD Biosciences 557703) was used for negative gating. Cells were washed three times with PBS 1% BSA then resuspended in PBS. Flow cytometry was performed with a FACS Melody cytometer (BD Biosciences) and data were analyzed with FlowJo V10.8 (BD Biosciences).

Confocal imaging

CK14 stained cells were observed using a confocal microscope (Zeiss LSM800) with ZEN Black (Zeiss 2011) software to create a z-stack image for the full thickness of the epithelial culture. Orthogonal projections were processed in ImageJ software.

Supplementary Figure legends

Supplementary figure 1: SOX17 expression of epithelial cells extracted using either thermolysin or dispase. *Symbols ○, ● , ●, ⊗ represent 22-, 28-, 41-, and 58-year-old donors. N=4. Statistical differences were calculated using ratio paired t-tests.*

Supplementary figure 2: **Characterization of extracted epithelial cells using dispase II.** *From the distal or proximal fossa navicularis (FN D, FN P) or the spongy urethra (SU), cells were compared to vaginal epithelial cells (Va) and bladder epithelial cells (B). Cells were stained with Hoechst (blue), cytokeratin 14 (CK14, green). Confocal slices of the basal and apical sections, as well as orthogonal projections are presented, demonstrating the apical location of CK14. Scale bars represent 50 µm.*

Supplementary figure 3: Immunofluorescence image of the vaginal mucosa. *Staining of Hoechst (blue) and cytokeratin 10 (CK10, red). Scale bar represents 200 µm.*

Supplementary Tables

Supplementary table 1: P-values of differences in colony forming units (CFU) of epithelial cells extracted with thermolysin or dispase. N=12 (4 donors, 3 region-paired biopsies per donor). Statistical differences were calculated using ratio paired t-tests.

**CFU**	P1	P2	P3	P4
total	0.8266	0.033	0.0506	0.024
holoclones	0.0097	0.0274	0.8341	0.7209
meroclones	0.0765	0.4681	0.4216	0.1599
paraclones	0.6816	<0.0001	<0.0001	<0.0001

Supplementary table 2: P-values of differences in yield at P-1 or P0 for fibroblasts digested in collagenase with (+) or without (-) elastase for 4 or 20 hours. N=4, n=3. Statistical differences were evaluated using a 2-way ANOVA followed by a Tukey's test.

		**P-1**			**P0**		
		**-**	+		**-**	+	
		20h	4h	20h	20h	4h	20h
-	4h	0.8871	0.29	0.7678	0.9659	0.0357	0.9554
	20h		0.0746	0.9947		0.0114	0.7622
+	4h			0.0432			0.1094

Supplementary table 3: P-values of differences in yield at P-1 for epithelial cells and fibroblasts extracted from 3-, 4-, or 6-mm biopsy punches. Absolute and surface normalized yields are presented. N=16 (4 donors, 4 region-paired biopsies). Statistical differences were evaluated using a 1-way ANOVA followed by a Tukey's test.

	**epithelial cells**				**fibroblasts**			
	absolute		normalized		absolute		normalized	
	4 mm	*6 mm*	*4 mm*	*6 mm*	*4 mm*	*6 mm*	*4 mm*	*6 mm*
3 mm	0.0052	0.0001	0.9988	0.7411	0.0002	<0.0001	0.3442	0.3027
4 mm		0.0012		0.8311		0.0003		0.1205
